# Acute and delayed toxicity from co-ingestion of methylene chloride and methanol

**DOI:** 10.1080/24734306.2019.1685222

**Published:** 2019-11-12

**Authors:** Todd A. Jaffe, Edward W. Boyer, Timothy B. Erickson, Heather Studley, Bryan D. Hayes, Peter R. Chai

**Affiliations:** a Harvard Affiliated Emergency Medicine Residency at Massachusetts General Hospital and Brigham and Women’s Hospital, Boston, MA, USA;; b Division of Medical Toxicology, Department of Emergency Medicine, Brigham and Women’s Hospital, Harvard Medical School, Boston, MA, USA;; c Behavioral Science Research Program, The Fenway Institute, Boston, MA, USA;; d Harvard Humanitarian Initiative, Harvard University, Cambridge, MA, USA;; e Clinical Pharmacist, Emergency Medicine and Toxicology, Massachusetts General Hospital, Harvard Medical School, Boston, MA, USA;; f Koch Institute for Integrated Cancer Research, Massachusetts Institute of Technology, Boston, MA, USA;; g Department of Emergency Medicine, Brigham and Women’s Hospital, Harvard Medical School, Boston, MA, USA

**Keywords:** Methylene Chloride, methanol, Carbon Monoxide, case report

## Abstract

Methylene chloride is a volatile, chlorinated hydrocarbon and colorless solvent found in multiple industrial products including paint strippers, metal cleaners, automotive products, pesticides and aerosol containers. Occupational exposure to methylene chloride is reported in automotive technicians, painters, and other industrial workers with adverse health effects including gastrointestinal, neurological, as well as hepato-renal injuries. International Agency for Research on Cancer (IARC) classifies methylene chloride as a 2 A carcinogen. Through a series of reactions catalyzed by cytochrome P450 2E1 (CYP2E1), metabolism of methylene chloride leads to the formation of formyl chloride, and ultimately carbon monoxide (CO). Most reports of methylene chloride toxicity are due to dermal and inhalational exposure in occupational settings. Ingestion of methylene chloride is uncommon, yet can lead to significant toxicity and prolonged CO toxicity. Methylene chloride is frequently formulated with methanol; individuals who intentionally ingest methylene chloride can experience concomitant methanol toxicity. We present a case of acute ingestion of paint stripper containing methanol and methylene chloride. We discuss the clinical presentation, key management decisions, relevant pathophysiology and biochemistry, as well as the clinical course and management.

## Introduction

**Figure 1.**
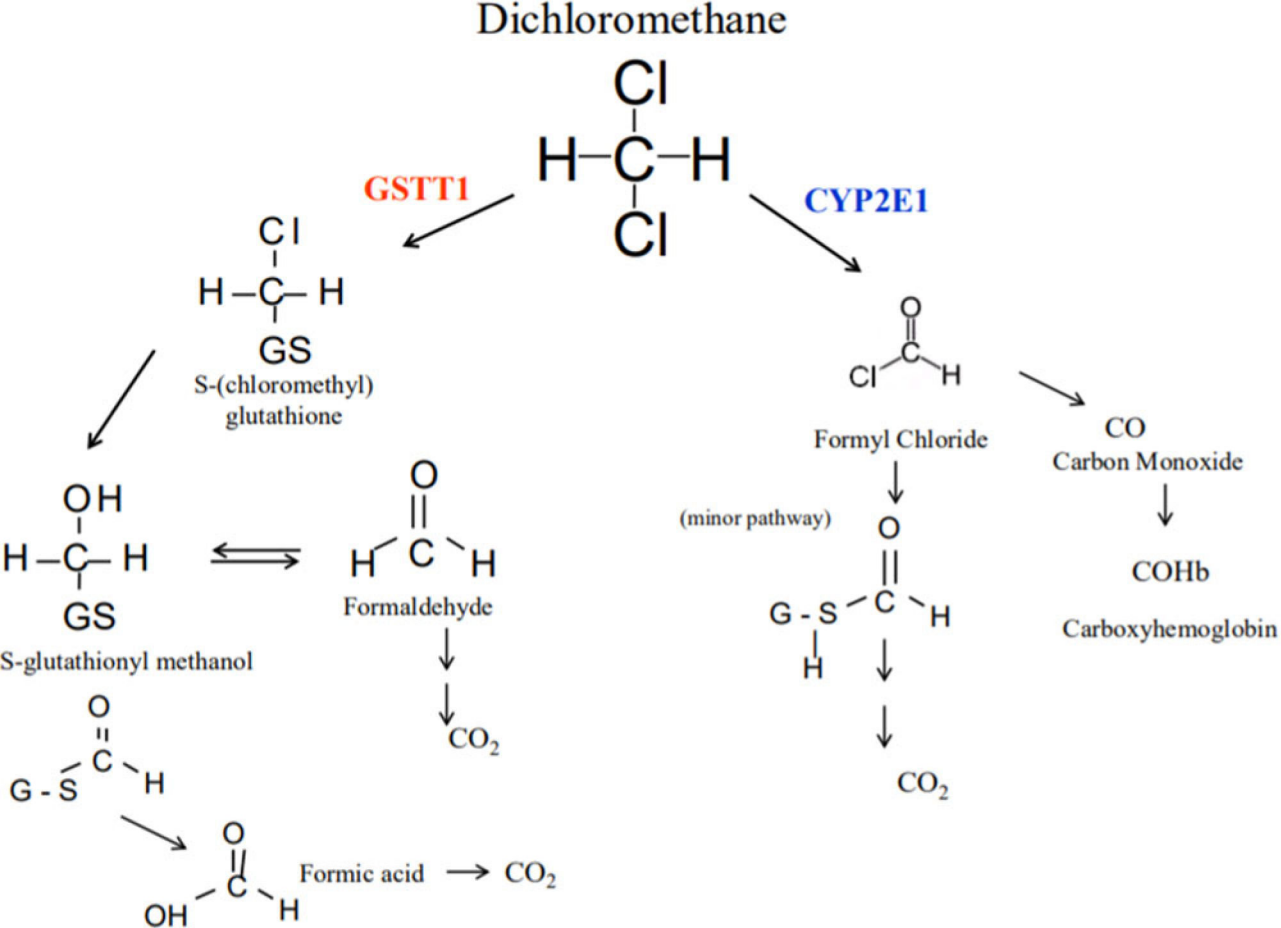
Methylene chloride metabolism. Methylene chloride metabolism includes two pathways. Through a reaction catalyzed by CYP2E1, methylene chloride becomes formyl chloride which is further metabolized to produce carbon monoxide. Glutathione S-transferase catalyzes a reaction in which methylene chloride forms S-(chloromethyl)glutathione. Replacement of chlorine leads to the formation of S-glutathionyl methanol, which can further rearrange to form formaldehyde or formic acid. Source, Public Domain: “Toxicological Review of Dichloromethane (Methylene Chloride).” Environmental Protection Agency Technical Report: EPA/635/R-10/003F. November 2011.

Methylene chloride is a volatile, chlorinated hydrocarbon and colorless solvent found in multiple industrial products including paint strippers, metal cleaners, automotive products, pesticides and aerosol containers [1]. Dermal and inhalational exposure to methylene chloride has occurred in painters, automotive technicians, and other industrial workers. Significant occupational exposure causes nausea and vomiting, depressed consciousness, pancreatitis, and hepatic and renal injury [2]. The International Agency for Research on Cancer classifies methylene chloride as a Group 2 A carcinogen; long-term exposure has been associated with biliary cancer and non-Hodgkin lymphoma [3]. Its major metabolic pathways include hepatic oxidation via cytochrome P450 2E1 (CYP2E1) and conjugation via glutathione-S-transferase theta 1 (GSTT1) [4,5]. Through the reaction catalyzed by CYP2E1, methylene chloride reacts with oxygen to form formyl chloride and then spontaneously rearranges to ultimately form carbon monoxide (CO) (Figure 1) [2,4].

**Figure 2.**
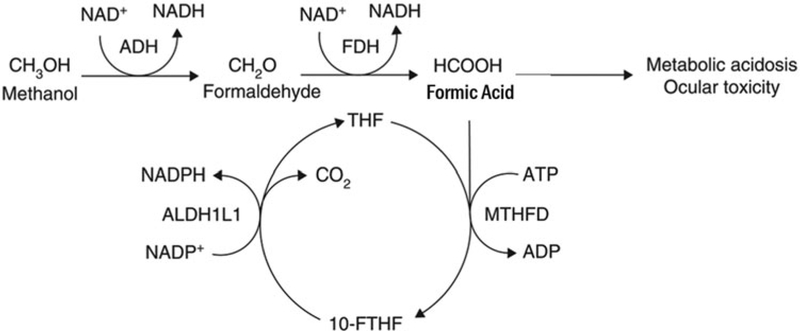
Methanol metabolism. Methanol metabolism includes conversion of methanol to first formaldehyde and then formic acid. The former reaction is catalyzed by alcohol dehydrogenase, whereas the latter is catalyzed by formaldehyde dehydrogenase. Formic acid is associated with many of the toxic effects of methanol including metabolic acidosis and ocular toxicity. Source: Marchitti SA, Brocker C, Stagos D, Vasiliou V. Non-P450 aldehyde oxidizing enzymes: the aldehyde dehydrogenase superfamily. Expert Opin Drug Metab Toxicol 2008;4:697–720. Permissions obtained.

Methanol is an organic solvent and toxic alcohol commonly found in industrial products including washer fluids, carburetor cleaners and fuel formulations [6]. In the body, alcohol dehydrogenase oxidizes methanol to formaldehyde; subsequently, aldehyde dehydrogenase oxidizes formaldehyde to formic acid (Figure 2) [7]. Methanol toxicity typically causes severe acidosis, altered mental status and blindness [8–13].

While occupational dermal and inhalation exposure to methylene chloride results in significant morbidity, there is a paucity of data describing intentional oral ingestion. We present the case of an acute co-ingestion of methylene chloride and methanol.

## Case presentation

A 72-year-old woman with a history of type 2 diabetes mellitus, chronic kidney disease, renal cell carcinoma, and depression presented to the emergency department (ED) after being found obtunded and cyanotic with agonal breathing at home. She was last seen well 4 h prior by family. EMS found a one-quart (946 milliliter) bottle of paint stripper half empty next to her on the floor. Paramedics described a “sweet solvent-like odor” emanating from her body. They administered intranasal naloxone 4 mg with no response, and then orally intubated the patient. Her home medications included amlodipine, levothyroxine, labetalol, metformin, rosuvastatin, losartan, mirtazapine, risperidone, clonazepam, buspirone, oxybutynin, acyclovir, and aspirin.

On arrival to the ED, the patient’s vital signs were: heart rate 56 beats per minute, blood pressure 101/51 mmHg, mechanically ventilated at 12 breaths per minute, oxygen saturation at 98% receiving 50% fraction-inspired oxygen. Laboratory values were significant for venous pH of 7.06, anion gap 16, white blood cell count 15,000 cells/mm^3^, potassium 7.0 mmol/L, lactate 3.2 mmol/L, and lipase 1,899 units/L (Table 1). Salicylates and acetaminophen were undetectable. Providers accessed the paint stripper’s material safety data sheet (MSDS), which revealed that the product contained 60–100% concentration of methylene chloride and 7–13% concentration of methanol. The initial serum carboxyhemoglobin (CO-Hgb) was 8.2%, and the patient was referred to a facility with hyperbaric therapy.

She received two grams intravenous calcium gluconate, 10 units regular insulin, 25 grams dextrose, IV sodium bicarbonate 8.4% 200mEq, and nebulized albuterol to address her hyperkalemia. Her ventilator settings were adjusted to a FiO_2_ of 100% in order to increase dissociation of carboxyhemoglobin. Additional laboratory data revealed a serum methanol concentration of 168 mg/dL. She received 15 mg/kg fomepizole intravenously, was admitted to the intensive care unit (ICU) and started on hemodialysis.

## Hospital course

**Figure 3.**
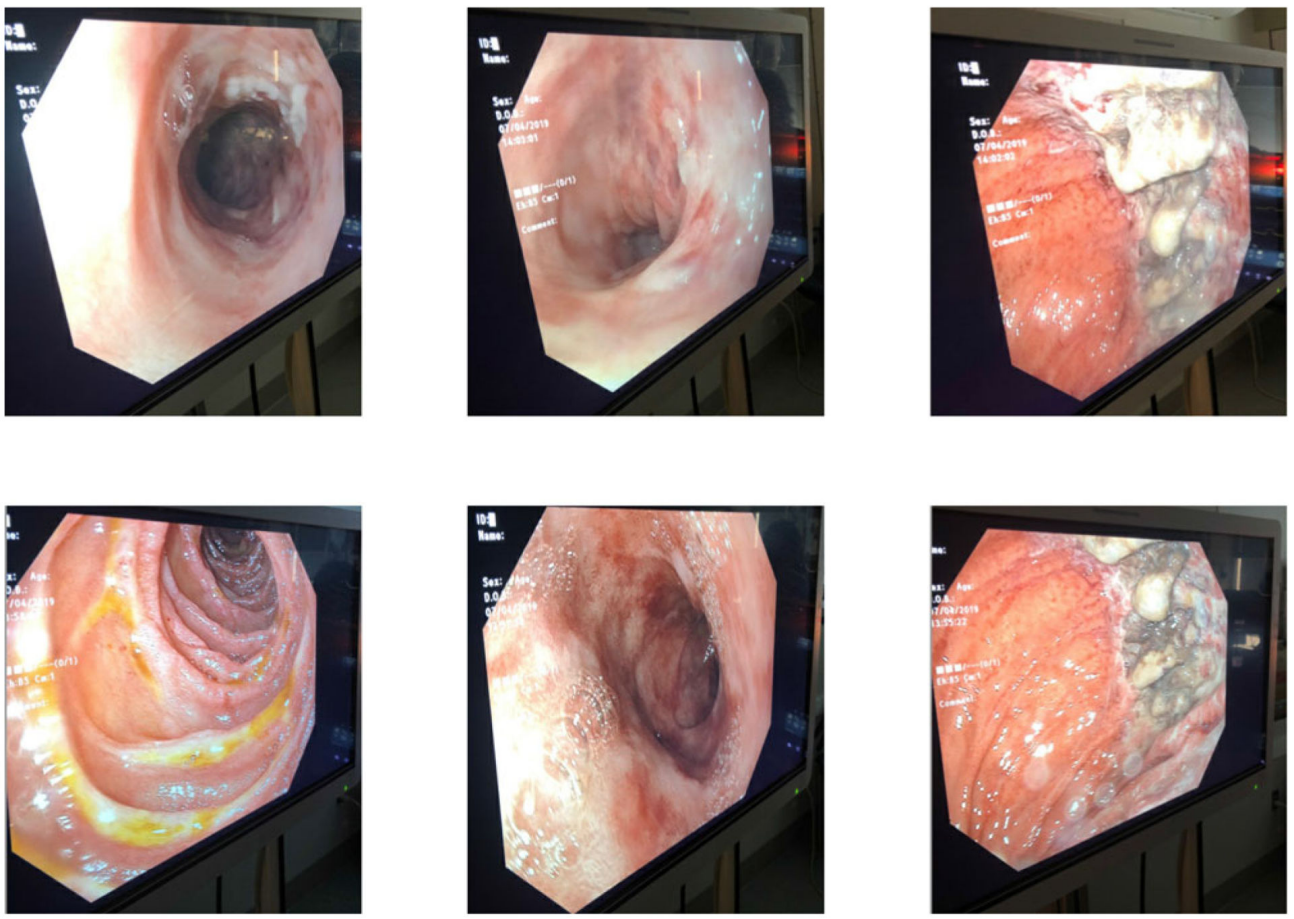
Endoscopic images from the patient’s electronic medical record. Obtained with patient permission.

Eight hours after presentation, her carboxyhemoglobin concentration decreased from 8.2% to 2%, with ultimate nadir at 0.7%. She was maintained on 100% FiO_2_ and hyperbaric oxygen therapy was deferred. She received eight hours of hemodialysis followed by continuous veno-venous hemofiltration (CVVH) for twelve hours. Antidotal therapy consisted of fomepizole 15 mg/kg once, followed by 10 mg/kg every 4 h during hemodialysis and 10 mg/kg every 8 h while receiving CVVH until serum methanol concentration was undetectable. She remained intubated for 80 h, during which she underwent magnetic resonance imaging (MRI) of the brain and esophagogastroduodenoscopy (EGD) due to concerns for caustic injury after the ingestion. MRI of the brain was normal, but EGD demonstrated eroded and ulcerated mucosa in the greater curvature of the gastric body and multiple nonbleeding jejunal erosions without evidence of bleeding (Figure 3). On day three of admission, she developed Proteus mirabilis pneumonia that was treated with cephalosporins. She was extubated on day four and subsequently transferred to a psychiatric facility.

## Discussion

Dual poisonings from a single exposure—in this case, methanol and carbon monoxide from an isolated paint stripper ingestion—are uncommon events that can baffle even seasoned clinicians. Clinical data regarding ingestion of methylene chloride are lacking since most clinical experience is in the setting of dermal or inhalational routes of exposure. This case is interesting, therefore, because of the lack of clinical data on the production of carbon monoxide after intentional methylene chloride ingestion. Emergency medicine physicians should consider investigating access and exposure to household cleaning agents and industrial chemicals in individuals with intentional ingestions who may present with headaches, altered mental status, or persistent hypoxemia.

Methylene chloride may be co-formulated with methanol; ingestions of methylene chloride, therefore, warrant evaluation for concomitant methanol poisoning. Rapid access to poison control center data as well as material safety data sheets (MSDS) are important resources for clinicians to discover potential toxic coingestants. Given methylene chloride’s toxic nature, the Environmental Protection Agency has recently banned the consumer sale of paint removers with methylene chloride [14]. Yet, older products with high methylene chloride concentrations remain prevalent as indicated in this case.

The few reports describing intentional methylene chloride ingestion are notable for striking severity and duration of poisoning. For example, one case describes a patient with a carboxyhemoglobin concentration as high as 35%; the patient’s concentration slowly decreased throughout her time in the hospital, however she died on the ninth hospital day [15]. This patient’s carboxyhemoglobin concentration at 120 h after ingestion stabilized at 9%. Management of oral methylene chloride ingestion includes administration of high levels of oxygen; two reports describe the utility of hyperbaric oxygen for acute methylene chloride inhalation exposure in obtunded patients with a carboxyhemoglobin concentration of 13.0% and 11.5%, respectively; each patient had neurologic improvement [16]. Although formal data are lacking, review of the medical literature suggests that mortality from methylene chloride poisoning, even among hospitalized patients, can be as high as 22% [15–18].

Our patient developed gastrointestinal injury, yet the exact mechanism of GI injury in methylene chloride remains largely unknown. One explanation is that the compound has a direct irritating effect on gastro intestinal mucosa as methylene chloride itself is a neutral molecule. Another possibility is that other chemicals co-formulated with methylene chloride are responsible for gastric mucosal irritation. Case studies have also described significant gastrointestinal sequelae from methylene chloride ingestion. In one case series of six patients, all six patients endorsed gastrointestinal symptoms including nausea and/or vomiting, and three underwent endoscopy. Two of the endoscopies demonstrated substantial esophagitis and gastritis [15]. Our patient also had evidence of pancreatitis with a significantly elevated lipase on presentation. Acute pancreatitis has been reported in both methylene chloride and methanol ingestion [7,15,19,20].

The patient presented with an elevated methanol concentration at 168 mg/dL and underwent treatment with both fomepizole and hemodialysis. Given the patient’s significantly elevated concentration and concern for delayed toxicity, targeted antidotal therapy was started soon after transfer. Guidelines from the American Academy of Clinical Toxicology recommend antidotal therapy with methanol concentrations above 20 mg/dL or strong clinical suspicion with two of the following: arterial pH <7.3, serum bicarbonate <20 mEq/L, or osmolal gap >10 mOsm/kg with a loading dose of fomepizole 15 mg/kg commonly referenced [6,21]. Regarding hemodialysis, guidelines from the Extracorporeal Treatments in Poisoning Workgroup (EXTRIP) recommend hemodialysis for severe metabolic acidosis with a pH <7.15 as well as coma, seizures, significantly elevated concentrations, as well as visual complaints in the setting of methanol ingestion [22]. Recommendations for concomitant treatment of methanol ingestion with hemodialysis, continuous veno-venohemofiltration (CVVH) and fomepizole may vary [21]. The manufacturer’s dosing instructions for fomepizole during hemodialysis, for example, recommends administering doses of the medication every four hours; unfortunately, the optimal dosing regimen during CVVH is unknown [23]. Although less studied, some recommendations have included decreasing the dosing interval for fomepizole to every eight hours during CVVH [24]. Our patient received the dosing indicated for hemodialysis, and after twenty hours of treatment, had no detectable methanol in the blood.

Overall, this case describes significant methylene chloride ingestion leading to carbon monoxide poisoning that was treated with 100% oxygen therapy in addition to fomepizole and hemodialysis for concurrent methanol toxicity. Supportive care, including intubation for airway protection, IV fluids, and close monitoring, contributed to the survival of this patient. This case highlights the importance of recognition of atypical sources of self-injury and the importance of MSDS access as a data source for industrial and household compounds. This also illustrates the importance of considering not only parent compounds when managing an acute overdose, but any potential delayed, and often more deleterious, effects from the by-products or toxic metabolites produced.

## Figures and Tables

**Table 1.** Selected laboratory values of patient.

	Time After Presentation (Hours)			
	0	4	8	10
Sodium (mmol/L)	135	136	144	144
Potassium (mmol/L)	7.0	6.7	5.2	3.6
Chloride (mmol/L)	111	108	114	112
Carbon Dioxide (mmol/L)	11	8	10	17
Anion Gap	13	20	20	15
BUN (mg/dL)	48	47	48	30
Creatinine (mg/dL)	1.01	1.12	1.09	0.56
Glucose (mg/dL)	209	352	395	163
AST (SGOT) (U/L)	84	91	129	–
ALT (SGPT) (U/L)	28	27	44	–
Lactic Acid (mmol/L)	3.2	2.6	1.7	–
Lipase (U/L)	1,899	–	–	–
Serum Osmolality (mosm/kg H_2_0)	^−^	380	^−^	^−^
VBG pH	7.06	7.11	–	–
VBG pCO2 (mmHg)	39	31	–	–
VBG HCO3^−^ (mmol/L)	11	10	–	–
ABG pH	–	–	7.07	7.35
ABG pCO2 (mmHg)	–	–	21	25
ABGp p02 (mmHg)	–	–	327	353
ABG HCO3^−^ (mmol/L)	–	–	6	14
MetHgb %	–	–	0.1	–
Carboxyhemoglobin %	8.2	4.5	2	0.7
Methanol (mg/dL)	–	168	154	28 (at 14h)

## References

[1] Phillips JA. Methylene Chloride. Workplace Health Saf 2018;66(2):108. 29053053 10.1177/2165079917736319

[2] Toxicological Profile for Methylene Chloride. Agency for Toxic Substances and Disease Registry 9 2000. 38252765

[3] IARC Working Group on the Evaluation of Carcinogenic Risk to Humans. Some Chemicals Used as Solvents and in Polymer Manufacture. Lyon (FR): International Agency for Research on Cancer; 2017 (IARC Monographs on the Evaluation of Carcinogenic Risks to Humans, No. 110.) DICHLOROMETHANE. 31829531

[4] Bos PM, Zeilmaker MJ, van Eijkeren JC. Application of physiologically based pharmacokinetic modeling in setting acute exposure guideline levels for methylene chloride. Toxicol Sci 2006;91(2):576–585. 16569727 10.1093/toxsci/kfj176

[5] Barry KH, Zhang Y, Lan Q, Genetic variation in metabolic genes, occupational solvent exposure, and risk of non-hodgkin lymphoma. Am J Epidemiol 2011;173(4):404–413. 21228414 10.1093/aje/kwq360 PMC3032803

[6] Kraut JA, Mullins ME. Toxic Alcohols. N Engl J Med. 2018;378(3):270–280. 29342392 10.1056/NEJMra1615295

[7] Marchitti SA, Brocker C, Stagos D, Non-P450 aldehyde oxidizing enzymes: the aldehyde dehydrogenase superfamily. Expert Opin Drug Metab Toxicol. 2008;4(6):697–720. 18611112 10.1517/17425250802102627 PMC2658643

[8] Cursiefen C, Bergua A. Acute bilateral blindness caused by accidental methanol intoxication during fire “eating”. Br J Ophthalmol. 2002;86(9):1064–1065. 12185141 10.1136/bjo.86.9.1064 PMC1771266

[9] Methanol toxicity. Agency for Toxic Substances and Disease Registry. Am Fam Physician 1993;47:163–171. 8418579

[10] Martinasevic MK, Green MD, Baron J, Folate and 10-formyltetrahydrofolate dehydrogenase in human and rat retina: relation to methanol toxicity. Toxicol Appl Pharmacol. 1996;141(2):373–381. 8975761 10.1006/taap.1996.0302

[11] Becker CE. Methanol poisoning. J Emerg Med 1983; 1(1):51–58. 6386968 10.1016/0736-4679(83)90009-4

[12] Jacobsen D, McMartin KE. Methanol and ethylene glycol poisonings. Mechanism of toxicity, clinical course, diagnosis and treatment. Med Toxicol 1986; 1(5):309–334. 3537623 10.1007/BF03259846

[13] Hovda KE, Hunderi OH, Tafjord AB, Methanol outbreak in Norway 2002–2004: epidemiology, clinical features and prognostic signs. J Intern Med 2005;258(2):181–190. 16018795 10.1111/j.1365-2796.2005.01521.x

[14] EPA Bans Consumer Sales of Methylene Chloride Paint Removers, Protecting Public. United States Environmental Protection Agency; 2019.

[15] Chang YL, Yang CC, Deng JF, Diverse manifestations of oral methylene chloride poisoning: report of 6 cases. J Toxicol Clin Toxicol 1999;37(4): 497–504. 10465248 10.1081/clt-100102442

[16] Rioux JP, Myers RA. Hyperbaric oxygen for methylene chloride poisoning: report on two cases. Ann Emerg Med 1989;18(6):691–695. 2729697 10.1016/s0196-0644(89)80531-1

[17] Cabrera VJ, Farmakiotis D, Aggarwal V. Methylene chloride intoxication treated with hyperbaric oxygen therapy. Am J Med 2011;124(5):e3–4. 10.1016/j.amjmed.2010.12.010 21531220

[18] Roberts CJ, Marshall FP. Recovery after “lethal” quantity of paint remover. Br Med J. 1976;1(6000): 20–21. 10.1136/bmj.1.6000.20-a PMC1638237 1247717

[19] Hantson P, Mahieu P. Pancreatic injury following acute methanol poisoning. J Toxicol Clin Toxicol 2000;38(3):297–303. 10866330 10.1081/clt-100100935

[20] Zakharov S, Kotikova K, Vaneckova M, Acute Methanol Poisoning: Prevalence and Predisposing Factors of Haemorrhagic and Non-Haemorrhagic Brain Lesions. Basic Clin Pharmacol Toxicol 2016; 119(2):228–238. 26806851 10.1111/bcpt.12559

[21] Barceloux DG, Bond GR, Krenzelok EP, American Academy of Clinical Toxicology Ad Hoc Committee on the Treatment Guidelines for Methanol P. American Academy of Clinical Toxicology practice guidelines on the treatment of methanol poisoning. J Toxicol Clin Toxicol 2002; 40:415–446. 12216995 10.1081/clt-120006745

[22] Roberts DM, Yates C, Megarbane B, Recommendations for the role of extracorporeal treatments in the management of acute methanol poisoning: a systematic review and consensus statement. Crit Care Med 2015;43(2):461–472. 25493973 10.1097/CCM.0000000000000708

[23] Inc PL. PRODUCT MONOGRAPH: Including Patient Medication Information, Antizol. 2017 Version 6.0.

[24] McMartin K, Jacobsen D, Hovda KE. Antidotes for poisoning by alcohols that form toxic metabolites. Br J Clin Pharmacol 2016;81(3):505–515. 26551875 10.1111/bcp.12824 PMC4767193

